# The Impact of Cooperative Behavior between Social Organizations during the COVID-19 Pandemic Outbreak in Shanghai: A Simulation Approach

**DOI:** 10.3390/ijerph20021409

**Published:** 2023-01-12

**Authors:** Weipeng Fang, Changwei Qin, Dan Zhou, Jian Yin, Zhongmin Liu, Xianjun Guan

**Affiliations:** 1 School of Economics and Management, Tongji University, Shanghai 200082, China; 2 The Institute of Disaster Medicine Engineering of Tongji University, Shanghai 200082, China

**Keywords:** public health, COVID-19 pandemic prevention, cooperative relationship network, RSiena, stochastic actor-oriented model

## Abstract

In 2022, a new outbreak of the COVID-19 pandemic created considerable challenges for the Shanghai public health system. However, conventional prevention and control strategies, which only rely on formal organizations, inefficiently decrease the number of infections. Thus, a multi-organization management mode is needed for pandemic prevention. In this paper, we applied a stochastic actor-oriented model (SAOM) to analyze how these social organizations cooperate with others and further identify the mechanism that drives them to create a reliable and sustainable cooperative relationship network from the perspective of social network analysis. The model allowed us to assess the effects of the actor’s attributes, the network structure, and dynamic cooperative behavior in RSiena with longitudinal data collected from 220 participants in 19 social organizations. The results indicated that the number of cooperative relationships increased during the pandemic, from 44 to 162, which means the network between social organizations became more reliable. Furthermore, all the hypotheses set in four sub-models were significant (t-ratio < 0.1, overall max t-ratio < 0.25, and e/s > 2). Additionally, the estimated values showed that four factors played a positive role in forming the cooperative relationship network, i.e., all except the “same age group effect (−1.02)”. The results also indicated that the social organizations tend to build relationships with more active actors in the community in every time period. This paper is of great significance regarding the innovation of public health system management and the improvement of Chinese grassroots governance.

## 1. Introduction

From March to June 2022, a new outbreak of COVID-19 occurred in Shanghai due to the rapid spread of the omicron variant, which created great challenges for the city’s public health system [1]. As the number of infections increased rapidly, the government created three risk zones (“closed-control area, semi-control area, and normal area”) in the city area to implement hierarchical management [2]. Theoretically, as Shanghai is one of the most developed cities with leading emergency response systems and high-level social governance in China, its government may have more experience in pandemic prevention. However, at the beginning of the pandemic, the results were far from satisfactory. Chairman Xi once said that “The community organizations (the street office, the resident’s committee, the volunteer groups, the self-organizations, etc.) are the key elements of an urban public safety management system which still play a role in pandemic prevention”. How can this be achieved? After the SARS pandemic, it was noticed that only a few community organizations could help the government to control the disease, which led to inefficient pandemic prevention. Thus, the more community organizations that participate in managing social affairs, the more efficient they will be, which is also known as a high-degree autonomous community [3,4]. However, in previous studies, the ways in which to build a such community have not been explained.

Compared to governmental organizations, community organizations are complex and closer to society [5,6]. Furthermore, during a pandemic, many emergency events are unexpected and uncontrolled, and we cannot rely solely on the government. Thus, to control the disease, it is important to create a multi-organization governance pattern and establish a sustainable collaborative relationship network between social organizations [7]. Several factors could affect collaborative behaviors. In social cognitive theory, behaviors are changed within individuals and the environment [8,9]. The former factors include personality [10], promotion [11], and mentality [12], and the latter include population characteristics [13], regulations [14], and cultural background [15]. In terms of the influence of objectives, several studies have focused on the characteristics of tasks, such as structure, content, and atmosphere [16,17]. In addition to the static influences mentioned above, the evolution of behaviors would also affect the formation of collaborative relationships. Several studies have shown that these relationships are connected through a rule in game theory. In other words, organizations cannot make a “completely rational” judgement (i.e., find potential partners) under such complex situations. Thus, they want to find a dynamic balance under a “limited rational” judgement instead [18]. The most common rules are direct reciprocity, indirect reciprocity, kin selection, network reciprocity, and group selection [19,20,21,22].

In summary, although influencing factors have been comprehensively identified in previous studies, less research has focused on the interaction between factors and cooperative relationship networks. In addition, in traditional studies regarding social network issues, changes in network ties are determined by the factors above, which cannot include dynamic changes in relationships and actors’ behavior simultaneously or examine how factors influence these changes [23]. To fill this gap, Snijders created a stochastic actor-oriented model (SAOM) from the perspective of dynamic social network analysis [24]. In this model, it is believed that the structure of networks is determined by the actors, which would be simultaneously affected by the existing structure (called the “endogenous effect”) and the attributes of actors (called the “exogenous effect”) [25]. Furthermore, the model has been extended to address the co-evolution of a network and behavior through a longitudinal dataset [26]. Therefore, the SAOM has been widely used in various fields. Boda et al. applied this model to identify how new students should be assigned into groups before entering a school, which could affect their classmate relationship network [27]. Wang et al. focused on the mutual influence of students‘ attributes and their behaviors on the formation of friendship networks in college [28]. Ploeg et al. focused on the way bullying works in elementary schools, and an age-dependent effect of bullying was proposed to control bullying [29]. Cao et al. tried to identify the evolution of project-based collaborative networks between enterprises [30]. Moreover, the model has also been applied in the study of a food safety collaborative supervision network, diffusion effects in transnational regulatory networks, the relationship between quitting and friendship networks, and the network dynamic of local industrial clusters [31,32,33,34]. Indeed, the SAOM model can be used to estimate a variety of social processes, and we could select various sociological theories to model our problem.

In this paper, we used the SAOM model to identify the mechanism of forming cooperative relationship networks between social organizations. Our objective was to ascertain how ties are created, maintained, or terminated by actors as well as how to build reliable and sustainable cooperative relationships. Furthermore, the model allowed us to simultaneously assess the effects of static factors and dynamic behaviors based on the collected longitudinal data. Therefore, with the application of this actor-oriented model, in this paper, we systematically discuss the internal logic of forming a cooperative relationship network among social community organizations, which is of great significance to the innovation of public health system management and the improvement of Chinese grassroots governance.

## 2. Methods

### 2.1. Basic Assumptions

The foundation of the simulation was based on four basic assumptions [25]. First, “continuous-time change” indicates that the evolution of the network tie or actor behaviors is based on continuous-time change, that is, the difference in a social network between two observations is the result of the superposition of many unobserved micro-changes at each time point. Second, “a Markov process” signifies a changing network, and the state of the network at a current moment depends on the previous state. Third, “an actor-based model” means that the actors determine the changes in network ties or behaviors (creation, endowment, or termination). However, this does not mean actors can change at will. The changes are made sequentially according to their attributes (such as position) or the influence of other actors. Fourth, “single change at onetime” means that at each interval, an actor can only make one change.

According to the basic assumptions, the evolution of the model mainly depends on two functions: the rate function (the speed by which the dependent variable changes) and the objective function (the primary determinant of the probabilities of changes) [35]:

(1) $$\lambda_{i}\left(\rho ,\alpha ,x,m\right)=\lambda_{i1}\lambda_{i2}\lambda_{i3}$$

The rate of change $\lambda_{i}\left(\rho ,\alpha ,x,m\right)$ can be defined as a product of dependent factors. $\lambda_{i1}$ denotes the dependence on the observation period, $\lambda_{i2}$ represents the dependence of actor covariates, and $\lambda_{i3}$ is the dependence of network attributes. ρ and α are the corresponding parameters of actor x in the period m [36]. It is noteworthy that modeling that begins with a constant rate function is applied in most cases [35].

(2) $$f_{i}\left(\beta ,x\right)=\sum_{k}\beta_{k}S_{ki}\left(x\right)$$

The objective function $f_{i}\left(\beta ,x\right)$ is a linear combination of the effects, where $\beta_{k}$ is the corresponding parameters of effects $S_{ki}\left(x\right)$ based on the observed longitudinal data. If $\beta_{k}$ > 0, the direction of network evolution is consistent with the effect; if $\beta_{k}$ = 0, the effect has no influence; if $\beta_{k}$ < 0, the evolution direction is opposite to the effect [37]. In addition, it should be noted that $\beta_{k}$ is the log-probability of these effects, which represents the contribution to the decision in the next time period [35]. The effect with higher parameters would contribute more to the decision of actors (creation or endowment ties).

To ensure the model meets the basic assumptions, we calculate the Jaccard index to estimate the overall network changes:

(3) $$J=\frac{N_{11}}{N_{11}+N_{10}+N_{01}}$$

where J is the value of the Jaccard index, and experience has shown that this value should not be less than 0.3 [38]. $N_{11}$ represents the number of existing ties in the two observation waves, $N_{01}$ is the number of newly created ties, and $N_{10}$ is the number of terminated ties.

### 2.2. The Procedure of Stochastic Actor-Oriented Model

**Figure 1:**
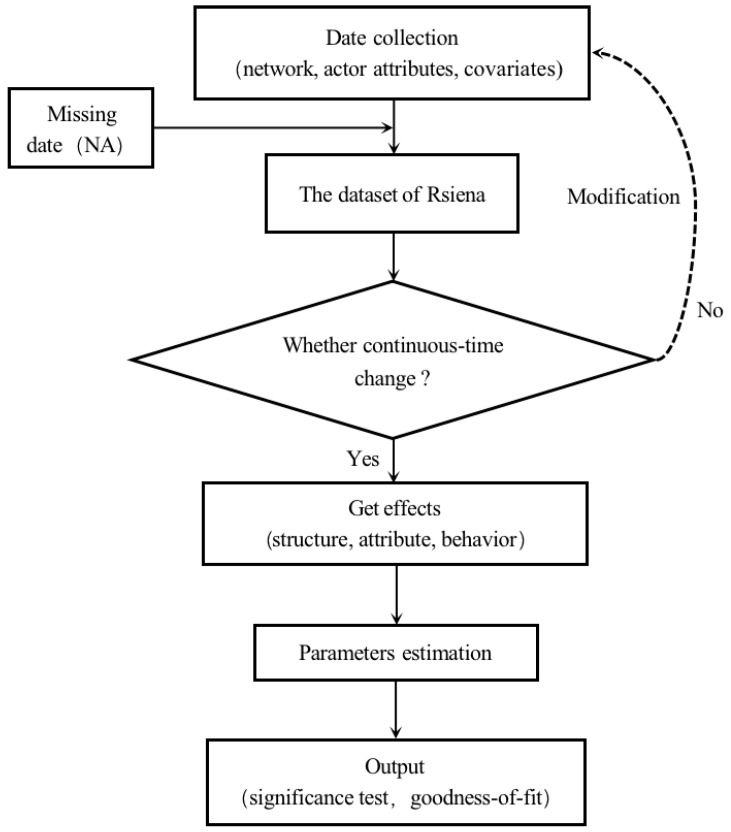
The procedure for the stochastic actor-oriented model (SAOM).

To model the evolution of cooperative relationship networks between social organizations during the COVID-19 pandemic, we applied a stochastic actor-oriented model (SAOM) with Simulation Investigation for Empirical Network Analysis (Siena) in the system RStudio (RSiena version 1.3.9). The procedure is shown in Figure 1.

### 2.3. Measures

#### 2.3.1. Cooperative Relationship Network

Using the cooperative relationship data we obtained from social participants, we established an undirected cooperative relationship network represented as a binary matrix (1 = the presence of a cooperative relationship; 0 = no relationship). It is noteworthy that the number of actors was dynamic during the COVID-19 pandemic, which may have caused some data to be missed in a specific observation wave. Additionally, to meet the data requirement of RSiena (the data should be a square matrix), if an actor was terminated in the next wave, we used the number “1” to represent the current relationship with other actors; if the actor is created in the next wave, we use the number “0” [35].

#### 2.3.2. Actor Covariate Attributes

There are two types of actor covariate attributes: the constant covariate and the dynamic covariate. The formal type means the value of one attribute would not change during the waves. We included the gender (1 = man; 0 = woman) and age (1 = below 20; 2 = from 21 to 30; 3 = from 31 to 40; 4 = 41 to 50; and 5 = above 50) of each participant. We also included the function of organizations to represent whether they were important in the community (1 = set goals for others; 2 = information transfer; 3 = executor). For the latter, the value was changeable, and we used the frequency of communication among social organizations to represent the actor’s behavior (1 = low; 2 = more or less; 3 = moderate; 4 = regularly).

## 3. Simulation

### 3.1. Data Collection

In this paper, an online questionnaire and face-to-face interviews were both conducted to collect data from 1 March to 1 June 2022. The participants were from 19 organizations that included governmental organizations, property companies, enterprises, hospitals, police stations, etc. (see Table 1). The questionnaire used in this paper was designed through a comprehensive literature review and modified according to the opinions of some sociologists. Additionally, to ensure the reliability of the data, the data collection process was carried out in two steps. The first step was the pre-research process, in which we invited several sociology experts and participants to answer the questionnaire. After a preliminary reliability analysis of the data, we proceeded to the second step: the formal research process. In this process, we distributed the questionnaire to all the participants. In total, we collected 140 questionnaires from 220 participants during the pandemic. As we received opinions from different participants in the same organization regarding the same question, we used the modal value to represent the overall response. After data processing, we acquired the cooperative relationship matrix and the value of the actor’s covariate attributes, which can be seen in https://github.com/Danielfangweipeng/SAOM.git (accessed on 1 January 2023).

### 3.2. Observation Waves

**Figure 2:**
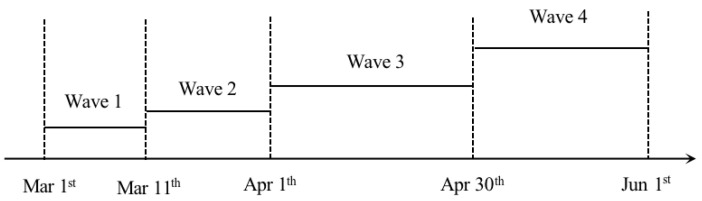
Observation waves during the pandemic.

According to the evolution of the COVID-19 pandemic in Shanghai, the observation time period could be separated into four waves, as shown in Figure 2. Wave 1 (1 March to 11 March) is the normalized prevention period, in which cooperative relationships hardly existed between social organizations; wave 2 (12 March to 1 April) is the spreading period, in which organizations such as hospitals and self-organization in the community began joining to control the pandemic; wave 3 (2 April to 30 April) represents the outbreak period of this pandemic. Numerous infections appeared in the community, which encouraged the government, self-organizations, hospitals, and other organizations to strengthen cooperation; wave 4 (1 May to 1 June) is the post-pandemic period. As the pandemic came under control, the cooperative behaviors of social organizations decreased gradually.

### 3.3. Research Hypothesis

Urban public health management is more difficult, especially when it is based on a dynamic cooperative relationship network that involves various social organizations. Actors would choose their potential partners based on the attributes and the structure of the network. In this paper, five hypotheses are proposed to measure the evolution mechanism of cooperative relationship networks during the COVID-19 pandemic in Shanghai.

#### 3.3.1. Core Organizations

The core organizations of a community have rich emergency supplies and firsthand information regarding the pandemic. On the one hand, they may be the managers of other organizations. On the other hand, the core organizations can obtain resources from others. Thus, the core organizations become the ideal cooperative partner of self-organizations and others. Moreover, if an organization has good relationships with the core organizations, it would also have more opportunities to create cooperation with others. Thus, we hypothesize that:

**H1.**  *The core organizations are active in the network and tend to create more ties with other actors.*

#### 3.3.2. Network Structure

As mentioned above, the evolution of a cooperative relationship network would be affected by the structure, for example, the transitive triads, betweenness, reciprocity, etc. [36]. As we focus on an undirected network in this paper, the transitive triads effect should be included, which can be regarded as generalized reciprocity and contribution to the network closure. For example, if actor A is the cooperative partner of actor B, and actor B has a relationship with actor C, then actor A tends to build a relationship with actor C. During the pandemic, governmental organizations are closed to the street office, but they are not familiar with the community organizations such as some self-organizations. Thus, they need to cooperate with the residents’ committee or self-organizations that are familiar with the street office. Additionally, in this pattern, the street office may be an intermediary in this cooperative relationship. Thus, we hypothesize that:

**H2.**  *Organizations tend to build transitive closure in the network.*

#### 3.3.3. Actor’s Basic Attributes

In previous studies, it has been shown that the gender and age of participants are important to build friendship relationships [34]. Additionally, it is also critical to create cooperative relationships between community organizations based on individual friendships. Thus, two hypotheses are proposed in the following:

**H3.**  *Social organizations would like to build relationships with others who are in the same age group.*

**H4.**  *Social organizations would like to build relationships with others who have a similar gender proportion.*

#### 3.3.4. Communication Behaviors

Communication behaviors between organizations are normally based on social trust, which can promote cooperative relationships. Individuals or organizations who have more similar communication behaviors are likely to become partners [39]. During the pandemic, organizations with frequent communication behaviors were beneficial for obtaining more supplies and information. Thus, other organizations would like to maintain cooperative relationships with them. We hypothesize that:

**H5.**  *Organizations tend to build cooperative relationships with organizations that have similar communication behaviors.*

### 3.4. Model Specification

To model the evolution mechanism of the network, four sub-models are estimated sequentially with different effects (see Table 2): Model 1 includes the “density effect” to predict the influence of the core organizations; Model 2 adds the “transitive triads effect” to show whether the community organizations tend to build network closure; Model 3 introduces the “same age group effect” and the “similarity gender proportion effect” to analyze the impact of an actor’s attributes; Model 4 includes the “similarity communication behaviors effect” to assess whether organizations with more similar communication behaviors tend to form relationships.

## 4. SAOM Results

### 4.1. Network Dynamics

**Figure 3:**
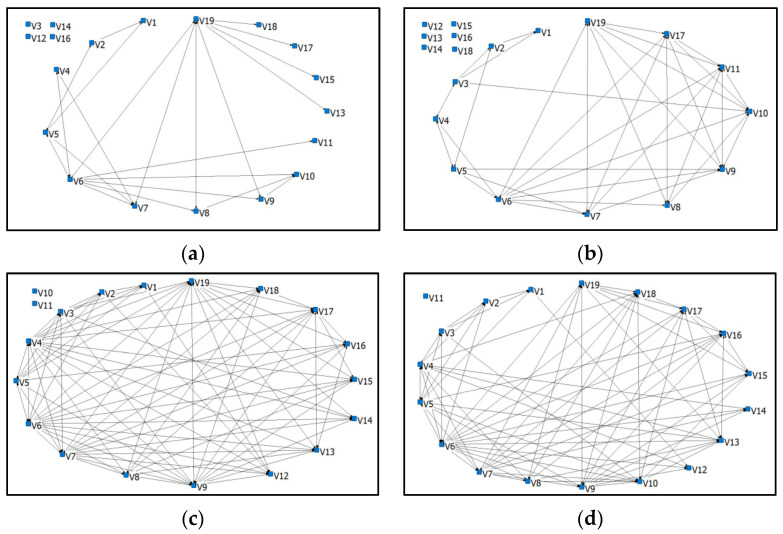
Cooperative relationship networks in four waves: (**a**) wave 1, (**b**) wave 2, (**c**) wave 3, (**d**) wave 4.

According to the data, the cooperative relationship network was gradually formed with the development of the COVID-19 pandemic, and the cooperation relationship increased significantly. Moreover, the number of actors also changed during the four waves, see Figure 3. In wave 1, as the disease was not widespread, the community was managed by governmental organizations such as the street office, and a few actors (V3, V12, V14, and V16) are not included. In wave 2, the number of people infected increased irregularly, and many social organizations started putting effort into controlling the pandemic. As it could be controlled in this period, some organizations (V12 to V16 and V18) still did not joined the network, but there was an obvious increase in the number of connections. In wave 3, the community was in a serious condition, and almost all the organizations (except V11; this actor was terminated) concentrated on preventing the pandemic. Additionally, the organizations tended to create relationships with others and form more stable cooperative relationship networks. Wave 4 represents the post-pandemic period, in which there was a decline in the number of infections, and medical and food supplies were back to normal (V10 and V11 were terminated). However, to avoid a repeated pandemic, social organizations need to remain in frequent communication.

The structural characteristics of the cooperative relationship network in four waves and its dynamics are presented in Table 3. Across each wave, the cooperative relationship network had 44, 72, 152, and 162 connections with other community organizations, which corresponded to network densities of 0.3056, 0.4615, 0.4967, and 0.5956 during wave 1 to 4, respectively. The structural stability displayed an obvious increase when calculating the average degree of the network at each wave, from 2.316 to 8.526. Moreover, the graph centralization also shows the apparent stability of the networks across four waves (35–40%). The Jaccard index is also listed in Table 3, the value of which was greater than 0.3, which meets the basic assumptions.

### 4.2. Network Dynamics

The objective of this paper was to determine whether actors’ attributes influence the formation of a cooperative relationship network and whether the network structure plays a role in creating connections between community organizations. Each sub-model assessed the effects simultaneously with network dynamics; the results are presented in Table 4. According to the manual, the corresponding value of each effect should have satisfied three requirements (if the effect was significant): (1) a convergence t-ratio less than 0.1; (2) an overall maximum convergence ratio less than 0.25; (3) an absolute value of Estimates/standard errors (e/s) greater than 2 [35].

In Model 1, we found that the formation and maintenance of cooperative relationships were significantly influenced by the core organizations. The estimated value was 0.80 (t-ratio < 0.1, e/s = 3.7), which means other organizations tended to create relationships with the core organizations, and thus, H1 was true. Additionally, it should be noted that the density effect was the default option in all the sub-modes according to SAOM. In Model 2, the estimated value of the transitive triads effect was 0.29 (t-ratio < 0.1, e/s = 3.40), which means actors in the dynamic network were more likely to build network closure, and the transmission of cooperative relationships between actors was also important; thus, H2 was true. The formation of the network was also impacted by actor covariates, including age and gender: namely, in Model 3, organizations that had a similar gender proportion were more likely to form cooperative relationships, the corresponding estimated value of which was 1.13 (t-ratio < 0.1, e/s = 14). Conversely, the estimated value of the same age group effect was −1.10 (t-ratio < 0.1, e/s = 9.71), which means organizations preferred to cooperate with different age groups. Thus, H3 was true, and H4 was false. Finally, in Model 4, we can see that organizations with similar communication behaviors tended to cooperate with others (t-ratio < 0.1, e/s = 2.12), and H5 was true. Overall, it was shown that the social organizations tended to build relationships with more active actors in the community in every time period.

**Figure 4:**
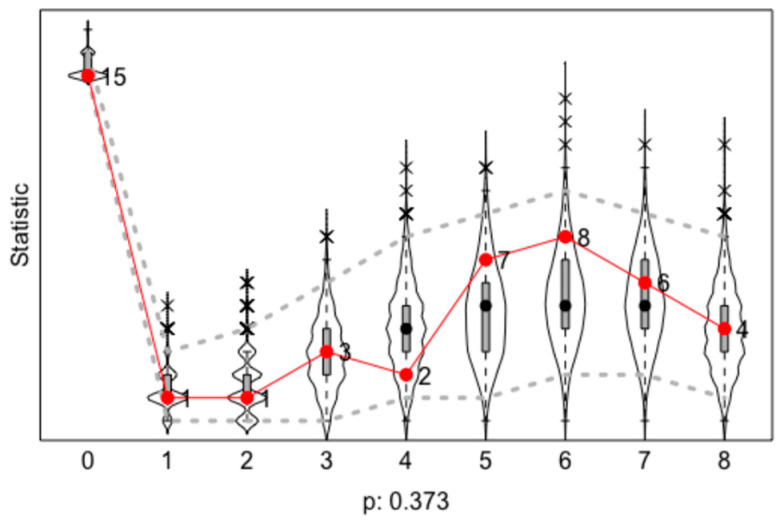
The result of model goodness-of-fit tests.

The results of the model goodness-of-fit tests are shown in Figure 4; from them, we learned that the observed values were included in the distribution of the iteration datasets, and the model fitting results were good (*p* < 0.05).

According to the results of the simulation, the SAOM model was used to identify the micro-mechanism and the evolution of the cooperative relationship network. Additionally, the model further quantitatively described the contribution level of influencing factors of actors’ behavior, which could not be considered in the traditional static social network analysis approaches. In addition, with the development of the COVID-19 pandemic, the number of actors changed during the four waves, which led to a changeable network structure. This dynamic process is also considered in the SAOM, whereas it cannot be considered in the traditional methods. Overall, it was shown that the SAOM model is suitable for dynamic cooperative relationship network analysis, and the results are reliable and scientific for the improvement of the efficiency of pandemic prevention in communities.

## 5. Highlights and Limitations

There are highlights of this study that are worth mentioning. First, the simulation data we used in the paper were collected from 220 participants in 19 social organizations who worked on the prevention of the COVID-19 pandemic in Shanghai. The data were first-hand and objective, which meant they could veritably represent the process of the formation of the cooperative relationship network during the four waves. Second, we used the SAOM model to identify the mechanism of creating cooperative relationships between social organizations in the community. By applying this approach, we were able to: (i) visualize the process of the cooperative relationship networks during the pandemic; (ii) identify the effects of actors’ attributes and network structure as the factors of forming relationships; and (iii) establish and verify five hypotheses, which means our findings are valuable. We included four types of potential factors that would affect cooperative behavior and used five effects to represent them in the SAOM, which allowed us to capture the intentions of social organizations.

It is important to recognize the sociological nature of these analyses and their limitations. The data sample we collected has a few weaknesses; for example, several cooperative relationships between organizations have been overlooked due to the data-processing method used. Additionally, to simplify the analysis, we used the participants’ (not all of them) preferences to represent the organization, which may have influenced the result of model estimation. In addition, although the model results presented in the paper support all the hypotheses, there are many extra effects that were not considered in this paper that may also play a role in the formation of a cooperative relationship network. Thus, if more detailed data are available in future research, more model effects could be added to this study. Finally, the co-evolution of networks and behavior dynamics should be considered in future research, and the mechanism of this network needs to be re-examined.

## 6. Conclusions

During the COVID-19 pandemic in Shanghai, the number of cooperative relationships between social organizations increased, but the cause for this increase is unclear. In this paper, we analyzed this phenomenon using data collected from various organizations and modeled the evolution of a cooperative relationship network in RSiena. We examined the effect of the network structure and the actors’ attributes and behavior in the model, which provided strong support for our hypotheses.

The results from the SAOM show that these factors mentioned above are important in the formation of a reliable and sustainable cooperative relationship network between social organizations. The results show that organizations having a similar gender proportion and communication behaviors highly influences the formation of relationships, but age is not a factor. On the contrary, organizations like to cooperate with those of different age groups who may have richer experience in pandemic prevention. Therefore, these findings suggest that a community may need diverse organizations, not only in social functions but also in attributes. In addition, the results also show that core organizations are active in creating cooperative relationships, which is consistent with empirical evidence. On the one hand, a pandemic prevention system in a community needs these core organizations to manage the community organizations. On the other hand, they play an intermediary role in the formation of relationships. Moreover, organizations that have high-frequency communication would provide strong support to the creation or maintenance of relationships with each other.

In this paper, we concentrated on identifying the mechanisms of the cooperative relationship network between community organizations, which is important for pandemic prevention and public health system management.

## Figures and Tables

**Table 1:** Description of organizations in the models.

Symbol	Name	Organization’s Function
V1	Shanghai municipal health commission	Publishing official information, setting goals, and management of other organizations
V2	Shanghai municipal center for disease control and prevention	Investigation, setting goals, and assigning tasks
V3	Police station	Security and medical transportation
V4	Street office	Medical and food supply, medical transportation, and management of community
V5	Hospital	Medical rescue and transportation
V6	Residents’ committee	Medical and food supply, management of community
V7	Owner committee	Food supply, management of residents
V8	Enterprise	Medical and food supply
V9	Manger of building	Management of residents
V10	Group buyer	Medical and food supply
V11	Leader of volunteers	Management of volunteers
V12	Property company	Healthcare, medical and food supply
V13	Healthcare group	Healthcare
V14	Transportation group	Medical and food transportation
V15	Digital data group	Broadcasting and posting information
V16	Purchasing group	Food supply
V17	Mental health counseling group	Psychological assistance
V18	Logistical support group	Disposing rubbish and transportation
V19	Medical supply management group	Medical supply

**Table 2:** Description of the effect included in the models.

Effect Name	Description
(Degree) density	Tendency to form relationships with other organizations
Transitive triads	Tendency to build closure in a cooperative relationship network
Same age group	Preference to create cooperative relationships with peers in the same age group
Similarity gender proportion	Preference to create cooperative relationships with peers with a similar gender proportion
Similarity communication behaviors	Preference to form relationships with organizations that have more similar communication behaviors

**Table 3:** Cooperative relationship network descriptive statistics.

Characteristic	Wave 1	Wave 2	Wave 3	Wave 4
Connections	44	72	152	162
Average of degree	2.316	3.789	8.000	8.526
Density	0.3056	0.4615	0.4967	0.5956
Centralization	35.29%	32.35%	49.67%	40.20%
Jaccard index	0.5854	0.6824	0.3298	

**Table 4:** The results of SAOM.

Sub-Models	Effect Types					
		Density	Transitive Triads	Similarity Gender Proportion	Same Age Group	Similarity Communication Behaviors
Model 1	Estimates	0.780				
	SD error	0.22				
	t-ratio	0.02				
	overall max t-ratio	0.02				
Model 2	Estimates	−0.25	0.29			
	SD error	0.30	0.00			
	t-ratio	0.0004	0.01			
	overall max t-ratio	0.08				
Model 3	Estimates	0.14	0.30	1.13	−1.10	
	SD error	0.36	0.09	0.43	0.38	
	t-ratio	−0.05	−0.08	0.03	−0.04	
	overall max t-ratio	0.07				
Model 4	Estimates	0.34	0.24	1.07	−1.02	1.46
	SD error	0.37	0.10	0.45	0.36	0.69
	t-ratio	0.04	0.06	−0.01	0.07	0.06
	overall max t-ratio	0.12				

## Data Availability

The data related to this paper can be found at https://github.com/Danielfangweipeng/SAOM.git, accessed on 1 January 2023.
